# Large SARS-CoV-2 Outbreak Caused by Asymptomatic Traveler, China

**DOI:** 10.3201/eid2609.201798

**Published:** 2020-09

**Authors:** Jingtao Liu, Jiaquan Huang, Dandan Xiang

**Affiliations:** Hubei University of Medicine, Shiyan, China (J. Liu);; Huazhong University of Science and Technology, Wuhan, China (J. Liu, J. Huang, D. Xiang);; Tongji Hospital, Wuhan (J. Huang, D. Xiang)

**Keywords:** respiratory infections, severe acute respiratory syndrome coronavirus 2, SARS-CoV-2, SARS, COVID-19, coronavirus disease, zoonoses, viruses, coronavirus, asymptomatic transmission, China, Heilongjiang

## Abstract

An asymptomatic person infected with severe acute respiratory syndrome coronavirus 2 returned to Heilongjiang Province, China, after international travel. The traveler’s neighbor became infected and generated a cluster of >71 cases, including cases in 2 hospitals. Genome sequences of the virus were distinct from viral genomes previously circulating in China.

Coronavirus disease (COVID-19), caused by severe acute respiratory syndrome coronavirus 2 (SARS-CoV-2), has spread rapidly around the world since the first cases were reported in late 2019 (*1*,*2*). Prior to April 9, 2020, Heilongjiang Province, China, had not reported a new COVID-19 diagnosis since March 11, 2020. On April 9, SARS-CoV-2 was diagnosed in 4 patients. By April 22, >71 persons had been infected. The likely origin of this cluster is an imported case from an asymptomatic traveler.

We collected and analyzed epidemiologic data published on the website of the Health Commission of Heilongjiang Province for April 9–23, 2020 (*3*). We defined confirmed COVID-19 cases as persons who tested positive for SARS-CoV-2 and had clinical symptoms. We defined asymptomatic carriers as persons without clinical symptoms who tested positive for SARS-CoV-2. We refer to case-patients by a letter for each family (A–Z, AA–ZZ), then by the assumed transmission generation (1–2), and finally in sequential order of exposure to SARS-CoV-2–positive persons in generations 1–3 (Figure) (*4*).

**Figure:**
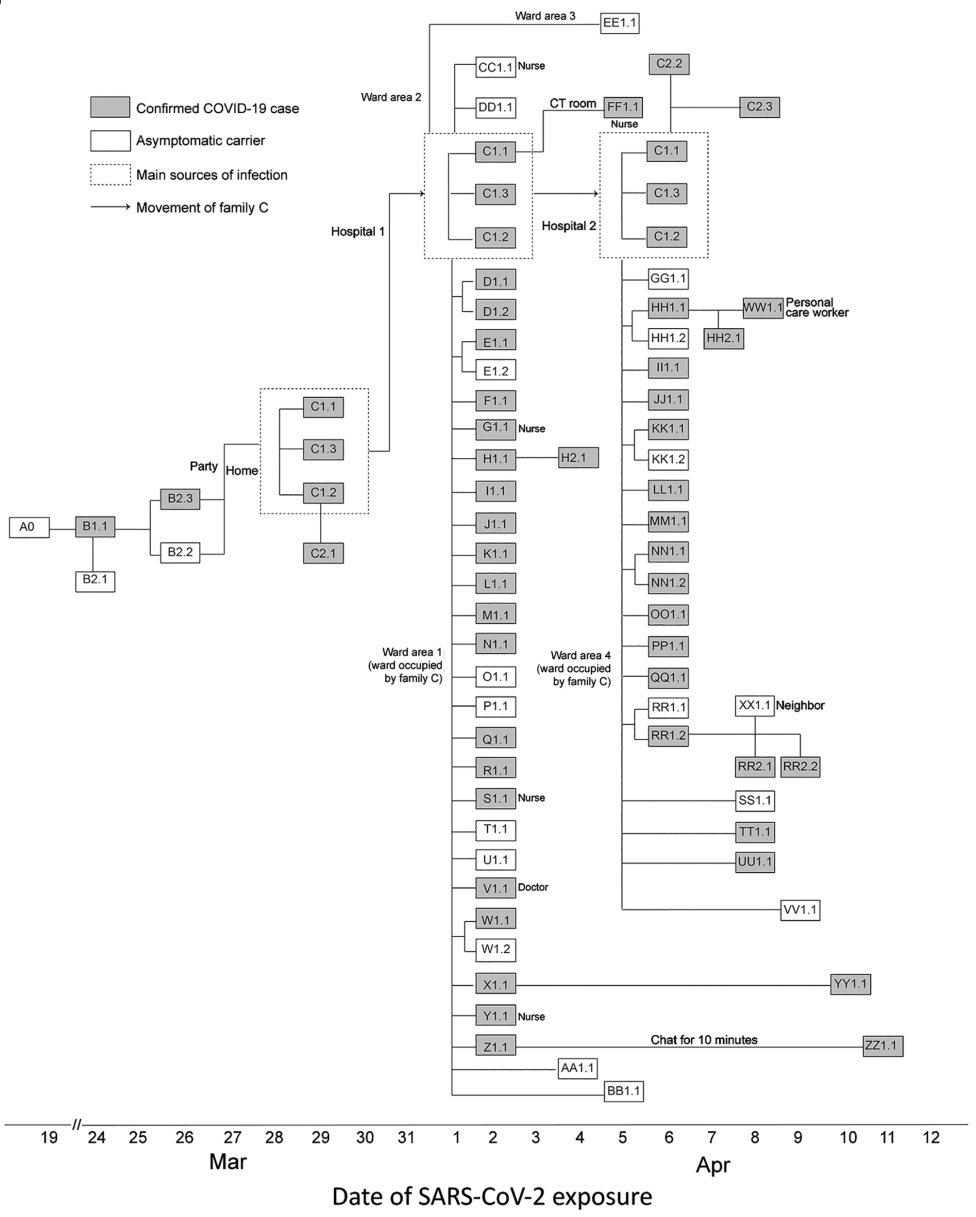
Timeline of exposure and connections between cases of severe acute respiratory syndrome coronavirus 2 (SARS-CoV-2) among persons in Heilongjiang Province, China. A0 returned from the United States on March 19, tested negative for SARS-CoV-2, and self-quarantined in her apartment and remained asymptomatic. However, SARS-CoV-2 serum IgM was negative and IgG was positive in later retests, indicating that A0 was previously infected with SARS-CoV-2 and likely was an asymptomatic carrier. B1.1, A0’s downstairs neighbor, likely became infected by using the elevator in the building after A0 had used it.

On March 19, 2020, case-patient A0 returned to Heilongjiang Province from the United States; she was asked to quarantine at home. She lived alone during her stay in Heilongjiang Province. She had negative SARS-CoV-2 nucleic acid and serum antibody tests on March 31 and April 3. 

Patient B1.1 was the downstairs neighbor of case-patient A0. They used the same elevator in the building but not at the same time and did not have close contact otherwise. On March 26, B1.1’s mother, B2.2, and her mother’s boyfriend, B2.3, visited and stayed in B1.1’s home all night. On March 29, B2.2 and B2.3 attended a party with patient C1.1 and his sons, C1.2 and C1.3.

On April 2, C1.1 suffered a stroke and was admitted to hospital 1. His sons, C1.2 and C1.3, cared for him in ward area 1 of the hospital. Patient C1.1 shared the same clinical team and items, such as a microwave, with other patients in the ward. On April 6, patient C1.1 was transferred to hospital 2 because of fever; C1.2 and C1.3 accompanied him. 

On April 7, patient B2.3 first noted symptoms of COVID-19. He tested positive for SARS-CoV-2 on April 9, the first confirmed case in this cluster. His close contacts, B1.1, B2.1, B2.2, and C1.1, subsequently tested positive for SARS-CoV-2 on April 9 or 10. Patient C1.1 was quarantined in hospital 2 when he tested positive on April 9. The epidemiologic investigation showed that none of these 5 persons had a history of travel or residence in affected areas with sustained transmission of SARS-CoV-2 during the 14 days before diagnosis, suggesting that SARS-CoV-2 came from contact with other persons. 

During C1.1’s admission at hospital 1, a total of 28 other persons, D1.1–BB1.1, were infected with SARS-CoV-2 in ward area 1. Because all patients in the ward could ambulate, 4 persons, CC1.1, DD1.1, EE1.1, and FF1.1, were infected in other wards and in the computed tomography room of hospital 1. Among hospital 1 staff, 5 nurses and 1 doctor were infected. In hospital 2, another 20 persons, GG1.1–VV1.1, were infected in the ward where C1.1 stayed (Figure).

On April 9, investigators also learned that A0, B1.1’s neighbor, had returned on March 19 from the United States, where COVID-19 cases had been detected. Investigators performed SARS-CoV-2 serum antibody tests on A0 on April 10 and 11. SARS-CoV-2 serum IgM was negative but IgG was positive, indicating that A0 was previously infected with SARS-CoV-2 (*5*,*6*). Therefore, we believe A0 was an asymptomatic carrier (*7*,*8*) and that B1.1 was infected by contact with surfaces in the elevator in the building where they both lived (*9*). Other residents in A0’s building tested negative for SARS-CoV-2 nucleic acids and serum antibodies.

On April 15, the Chinese Center for Disease Control and Prevention sequenced the entire genomes of 21 samples from the cluster. Viral genomes were identical in 18 cases and 3 other cases had a difference of 1–2 nucleotides, indicating that SARS-CoV-2 came from the same point of origin. The viral genome sequences from the cluster were distinct from the viral genomes previously circulating in China, indicating the virus originated abroad (*10*) and suggesting case A0 was the origin of infection for this cluster. 

All persons associated with this cluster, including those who lived in the same community and had close contact with SARS-CoV-2–positive patients or visited the 2 hospitals during April 2–15, were tested for SARS-CoV-2 nucleic acids and serum antibodies. As of April 22, 2020, A0 remained asymptomatic, and a total of 71 SARS-CoV-2–positive cases had been identified in the cluster.

Our results illustrate how a single asymptomatic SARS-CoV-2 infection could result in widespread community transmission. This report also highlights the resources required for case investigation and challenges associated with containment of SARS-CoV-2. Continued measures to protect, screen, and isolate infected persons are essential to mitigating and containing the COVID-19 pandemic.
